# An overview of the methodological aspects and policy implications of willingness-to-pay studies in oral health: a scoping review of existing literature

**DOI:** 10.1186/s12903-020-01303-3

**Published:** 2020-11-12

**Authors:** Navid Saadatfar, Mohammad Pooyan Jadidfard

**Affiliations:** Department of Community Oral Health, School of Dentistry, Shahid-Beheshti University of Medical Sciences, Shahid-Chamran Avenue, Evin, Tehran 19839 Iran

**Keywords:** Health service, Oral health, Dentistry, Willingness-to-pay, Discrete choice experiment, Patient preference

## Abstract

**Background:**

Demands for dental services seem to be beyond the capacities of most healthcare systems these days. Patient preferences have been increasingly emphasized to be considered in the joint decision-making process. Willingness-to-pay (WTP) is a recommended method for measuring the utility of health services; increasingly being used in recent decades. Taking these points into consideration, this article aims to provide an overview of the methodological aspects and policy implications of WTP studies in the field of oral health.

**Methods:**

The research was conducted in ISPOR, PubMed and Google Scholar databases. In addition, reference lists of included articles were checked to identify the relevant studies. All studies published were included that were in the English language and reported using WTP for oral health-related goods and services. A data-charting form was developed by a focus group discussion panel of seven experts to derive the main methodological aspects of WTP. Also, Core policy suggestions were categorized through thematic content analysis of the included papers.

**Results:**

The search strategy yielded 389 studies of which 52 were included. WTP studies in oral health show an increasing trend in global publications. The UK and Canada have a greater share in published material than in any other country. The dominant field of these researches is in restorative and prosthetic dentistry, and a wide range of different methodological aspects was documented. Policy suggestions were categorized in three main themes: (A) setting new tariffs or subsidizing the item, (B) provision of the item due to population preferences, and (C) improving literacy regarding the item.

**Conclusions:**

An urgent need for a common framework regarding the design of WTP studies in dentistry seems paramount. Some policy suggestions seem not to be applicable, perhaps due to insufficient familiarity of the researchers with the complexities of the public policymaking process.

## Background

In joint decision making between patient and health care providers, preferences of patients for proposed procedures are at least as important as clinical norms [1, 2]. On the other hand, demands for dental services are beyond the capacities of healthcare systems particularly in a majority of low and middle income countries [3].

Policymakers and health care managers must be informed about the different types of health care benefits perceived by patients, as well as factors influencing their services utility, in order to allocate optimal resources and generate favorable incentives within the healthcare systems [4, 5]. Patient valuations should include factors beyond service effectiveness, such as time span, discomfort, pain and anxiety [2].

“Willingness-to-pay” (WTP) is a systematic and trustworthy method in monetary terms to measure the benefit of a health care intervention [4]. WTP seeks to measure the preference strength of an individual for any desired intervention by calculating the maximum amount of money they would sacrifice [6]. This is considered the manifestation of “direct democracy” in public policymaking [7]. The technique is primarily seen as an aid to place monetary value on health care programs and to compare them particularly with programs beyond the health sector [8, 9]. WTP results can serve as the benefit wing of the economic evaluation (cost–benefit analysis) of a desired service, compared to other alternatives, which is strongly recommended as a critical input at the public level for decisions of allocation [10]. Therefore, WTP can help tailor dental treatment to individual patient valuations in a clinical practice, or help policymakers make informed decisions regarding resource allocation in the public sector and a priority setting across patient groups [8, 9].

WTP can be measured in two main ways: First, the “revealed preferences” approach which focuses on consumer behavior in the market and can be measured based on the information acquired from actual real market purchase of individuals and second, “stated preferences”; an indirect technique in which consumers are asked to explicitly state their WTP [11, 12]. “Contingent Valuation” (CV) is a WTP survey asking participants to state their maximum WTP for a hypothetical item [11].

WTP can be elicited through interview or questionnaire but to achieve a more valid result it is recommended to use the face-to-face interview method [13]. WTP is versatile and can be used for inquiring about a public service such as water fluoridation for a state, or it can be a non-public service such as tooth filling service provisions in a private clinic. There are several methods to elicit the WTP amounts: (a) the “open-ended” questions in which respondents are asked to freely state their maximum amount of WTP, (b) the “Take-It-Or-Leave-It” (TIOLI) in which respondents agree or disagree with one proposed value, (c) the “bidding game” which starts with a single bid and increases or decreases in accordance with the respondent agreement till the maximum WTP is reached, (d) the “payment cards” in which respondents decide among cards presenting various values (If the cards are presented randomly the technique is called “shuffled payment cards”), and (e) “payment scales” in which respondents should select a range of values which consist of their desired maximum WTP [14]. These techniques can be used alone or combined with another elicitation method. The “open-ended” questions may lead to inaccurate answers because of “strategic bias” (this occurs when respondents behave strategically to influence the provision or funding of the asked item instead of expressing their true WTP amounts) [15]. Although the “bidding game” provides a “market-like” situation, it could suffer from “starting-point” bias (when the first presented amount affects the true maximum WTP of respondents) [15]. Need for a larger sample size and being susceptible to “starting-point” bias are disadvantages of TIOLI [15]. Although the “payment cards” method may be affected by “range bias” (effect of the range of amounts printed on the cards on the WTP amounts) it may not be suitable to be used in rural areas. Regarding the oral health field, some authors have recommended the “shuffled payment card method” as the most appropriate method to be applied for WTP studies [16].

Johannesson and Meltzer have recommended that investigation of societal WTP for health care should be a research priority [17]. WTP has also been suggested as the most appropriate method to measure patient preferences in dental care programs; both in publicly funded health care programs and in private insurance based plans [18–20].

Since 1999 when the first WTP study in dentistry was published, the number of such studies dramatically increased globally over two decades. A similar trend in CV studies on general health has been documented over a broader time span [21]. Therefore, it seems to be the proper time to have a critical overview of the existing WTP studies in oral health care to search for any implication of the results for oral health policymaking, and in the future help undertake more robust practical research. Although a valuable critical review probed methodological aspects of WTP studies related to clinical services [22], in the current review both clinical and non-clinical studies are included. In addition, this study aims to conduct a scoping review on the existing oral health literature on WTP studies with a particular focus on policy implications and to examine areas of shortcomings in this field.

## Methods

We chose a scoping review methodology on WTP studies in the area of dentistry to explore all aspects of oral health services to derive diverse methodologies and policy implications. Our methods align with the 5-step methodological framework recommended by Arksey and O’Malley, albeit the first two steps are amalgamated [23]. The last search was done on March 15, 2020.

### Identification of research question and relevant research studies

The main question of this scoping review was: “What are the methodological attributes and policy implications in dentistry of the existing WTP studies?” Keywords were selected under two main concepts: Oral health [with the main keywords but not confined to them; “oral health”, “oral health care”, dentistry, “dental care”, “dental service(s)”] and willingness-to-pay [with the main keywords but not confined to them; “willingness-to-pay”, WTP, “contingent valuation”, “discrete choice experiment”, DCE, patients’ preferences, patients’ valuations and patients’ utilities]. Searches were conducted in PubMed, Google Scholar and the leading global International Society of Pharmacoeconomics and Outcomes Research (ISPOR) electronic databases with variations, and a combination of the keywords under two main aforementioned concepts. Reference lists of published oral health WTP articles were checked to identify relevant studies. The research question, search strategy and aim of the study were designed and discussed by both authors of the present study. Navid Saadatfar (NS) searched, removed duplicated articles, matched the obtained papers containing eligibility criteria, and then, extracted the data from the included papers. Mohammad Jadidfard revised the results and interpreted the data.

### Study selection

Potential studies published in English reporting WTP for oral health related goods and services were considered in this review including original articles, online available dissertations and official reports. No restrictions involving terms of study time, location or methodological aspects of WTP were considered. Studies were excluded eliciting preference of participants using methods other than WTP (e.g. “time-trade-off”) or studies using WTP without considering its effect on the oral health. Only reported article results of a dissertation were included in the review. If the data set was common in two or more articles, only one was included. Finally, two researchers independently reviewed all included studies.

### Data items and data charting process

A data-charting form was developed to assess oral health WTP studies by focus group discussion of a panel of seven experts including three from Dental Public Health; these members are practicing clinicians, two health economists, one from health policy and one health care management specialist. The checklist items included sample size, sampling and WTP elicitation methods, desired goods and services, factors affecting the amount of the stated WTP and policy recommendations. Sampling methods were categorized as ‘convenience sampling’ and ‘general population’. The goods and services sections were categorized as ‘public’ and ‘private’ items. An item was considered public when its use by others did not limit the availability to any other person and when individuals could not be excluded from its use. Factors affecting WTP were charted if the article reported any statistically significant association in this regard. A data charting form was calibrated before being used by the authors. This data charting form was pretested by five randomly selected articles and resulted in a satisfactory level of agreement between the authors. The data charting process was done independently by NS.

### Collating, summarizing and presenting findings

The methodological quality was not formally appraised as no specific checklist existed. Each paper was examined for any kind of policy implication inferred by the authors. A thematic content analysis method was used in order to identify the core policies in the documents. Contents of the documents were classified by an inductive-stepwise approach to the extract codes and final abstraction of the main themes. The included articles were examined and three final main themes (Core policies) were identified. All studies were investigated by both authors and any disagreements were resolved through discussion.

## Results

### Overview of WTP studies in oral health

**Fig. 1:**
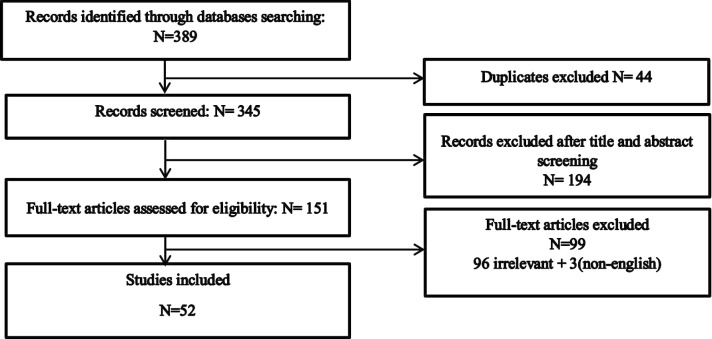
Inclusion diagram

**Fig. 2:**
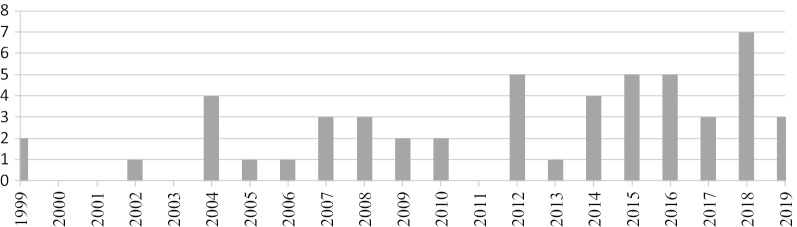
Number of published willingness-to-pay articles in oral health by year

The initial search resulted in identifying 343 articles using Google Scholar, 45 in PubMed and 1 in ISPOR and of which 52 were included in this review (48 papers and 4 theses and official reports). Finally, included articles consisted of 12 descriptive studies and 40 correlational studies (studies that seek to calculate any association between variables and WTP). A flowchart of the searched studies is shown in Fig. 1. Twenty-seven out of 52 articles (52%) were published following the onset of 2014. Figure 2 shows the frequency by year of all published oral health WTP studies included in this review from 1999 to the beginning of 2020. Three studies were executed simultaneously in two countries [24–26]; the UK and Canada published WTP studies in oral health more than any other country. Table 1 shows published WTP studies by continent and country.

**Table 1:** Number of willingness-to-pay studies in each continent and country

Continent and number of studies	Country	Number of studies	Europe 26 studies (47%)	UK	8
Asia 13 studies (23%)	KSA	3		Italy	5
	Hong Kong	3		Norway	3
	Iran	2		Finland	2
	Thailand	2		Bulgaria	2
	Singapore	1		Croatia	1
				Scotland	1
	India	1		Switzerland	1
	Philippines	1		Germany	1
North America 11 studies (20%)	Canada	8		Netherland	1
	USA	3		Sweden	1
Africa 2 studies (4%)	Kenya	1	Australia 1 study (2%)	Australia	1
	Tanzania	1	South America 2 studies (4%)	Brazil	2

(%) = proportion to weight of each continent in the existing dentistry WTP studies

Eight studies have elicited WTP for goods and services which were not commonly provided in the market at the time of those studies [2, 10, 27–32]. Twelve studies evaluated WTP for public services such as “universal dental insurance” or “water fluoridation” [26–29, 32–39], while 38 studies measured WTP for private services such as “root canal treatment” (RCT) or “implants”. One study assessed WTP for ‘waiting time before service utilization’ [40] and another study elicited WTP for different states of oral health [36]. Table 2 shows the frequency of WTP studies by service type. Items evaluated in each study, details about the methodological aspects and policy suggestions are shown in Table 3.

**Table 2:** Number of willingness-to-pay studies by each type of dental interventions

Field of study	Number
Restorative and prosthodontics	10
Oral health care schemes or insurance	9
Orthodontics	8
Combination of two or more fields	8
Preventive interventions	7
Oral medicine (anesthetic drugs or injection methods)	3
Special care (dentin regeneration, fear treatment, sonic toothbrush)	3
Endodontics	2
Periodontics	1
Oral surgery	1

**Table 3:** All studies included in the review (sorted by year)

	References	Country	Sample and elicitation method	Evaluated good or service	Main factors influencing WTP	Main policy recommendation/themes^a^
1	Matthews et al. [60]	Canada	42/Convenience sample + users/interview/bidding game	Periodontal therapy	Income	No policy suggestion was found
2	Dixon et al. [27]	UK	100/general population/interview/payment card	Water flouridation	–	Continue public education/(theme C)
3	Matthews et al. [6]	Canada	293/Patients + General Population/Computer-Based Tool (Questionnaire)/Bidding Game	Anaesthetic gel	Concern about dental pain and anxiety about needles/Insurance/Anxiety for gel	No policy suggestion was found
4	Pavlova et al. [47]	Bulgaria	990/General population/Interview/Payment intervals followed by open-ended/(WATP)	Dental check-up/filling or extraction of a tooth/Placement of dental prostheses	Family budget/Place of residence/Gender/Being chronically sick/Self-perceived health/Quantity of services used last year	Family budget Should remain the primary exemption criterion from patient payment obligations. etc./(theme A and B)
5	Birch et al. [2]	Canada	611/General population/telephone Interview/Open-ended	Dentin regeneration	Visit dentist regularly/Perceived need of tooth extraction/Insurance status	No policy suggestion was found
6	Halvorsen et al. [10]	Norway	62/Convenience sampling/Telephone interview/Open-ended	Dental fear treatment	Household income/Uncertainty/Perceived Benefits from dental and dental fear treatment/Change in dental health/capital/Treated surfaces	Supply of dental fear treatment should be subsidized/(theme A)
7	Smith et al. [48]	UK	188/General population + patients/Interview/Payment card	Orthognathic treatment	Incisor relation/Being patient	No policy suggestion was found
8	Ying et al. (Report) [61]	Hong Kong	257 Convenience sampling/Interview/Bidding game	RCT	Age/Education/Self-reported number of teeth possessed	No policy suggestion was found
9	Marvasti et al. [40]	USA	753/Convenience sampling/Questionnaire/Payment intervals	Waiting time	Income/Education/Actual waiting time/Medical experience/Gender/Age/Health insurance/Waiting location	No policy suggestion was found
10	Oscarson et al. [49]	Sweden	82/19 YO/General population/Interview/Binary valuation + open ended	Caries preventive Care	Caries risk/Housing	No policy suggestion was found
11	Atchison et al. [54]	USA	315/Convenience Sampling/Questionnaire/NM	Oral surgery	Income/Psychosocial predictors/Substance abuse/larger social network	No policy suggestion was found
12	Balevi and Shepperd [41]	Canada	40/Convenience sampling/Interview/Bidding	Crown, implant, bridge, removable partial denture	–	No policy suggestion was found
13	Tianviwat et al. [33]	Thailand	205/General population/Interview/Bidding game followed by open-ended question	Three dental services in two different settings	Care delivery setting/Income	More attention to establishing school-based services that Approach the same levels of effectiveness/(theme A)
14	Tianviwat et al. [34]	Thailand	205/General population/Interview/Bidding game followed by open-ended question	Prevention versus cure	Income/Age/Education/Service’s experience	Reduce the cost of using the sealant services to parents/(theme A)
15	Tuominen et al. [35]	Finland	156/Convenience Sampling/Questionnaire/NM	Helicopter ambulance service/MPR vaccination/Breast cancer screening/Hip Replacement/Dental check-up program for 7 YO	–	No policy suggestion was found
16	Esfandiari et al. [58]	Canada	36/Convenience sampling/Interview/Open-ended and yes-or-no question	Implant overdentures	Method of payment	Government program should be implemented To assist elders with the cost of denture Prostheses/(theme A)
17	Rosvall et al. [57]	USA	50/Convenience sampling/Questionnaire/Payment scale	Orthodontic appliances	–	No policy suggestion was found
18	Leung et al. [62]	Hong Kong	59/Convenience sampling/Interview/Bidding game	Anterior and posterior Implant	Gender/Education/Perceived need	No policy suggestion was found
19	Manickam et al. [42]	India	400/Convenience sampling/Questionnaire/NM	Anterior and posterior RCT	Age/Education/Income/Non vegetarian food habit	Government should improve literacy/(theme C)
20	Feu et al. [63]	Brazil	252/Convenience sampling/Interview/Payment scale	Orthodontic appliances	Socio-economic status/Age	No policy suggestion was found
21	Widstrom et al. [50]	Finland	704/General population/Postal questionnaire/Open-ended	Unexpected Dental expenses	High income/Subjective need for dental treatment	Benefit those with lower income and improve Quality of dental care for adults/(theme A)
22	Ethier et al. [52]	Canada	142/Convenience sampling/Interview/Bidding game	Prophylaxis of mucositis	–	No policy suggestion was found
23	Vermaire et al. [36]	Netherland	290/Convenience sampling/Questionnaire/Payment scale/(+ wti method)	Good state of oral health	Dmfs/Brushing time/Number of preventive visits/Dental hygiene burden/Dental hygiene knowledge/Importance to their Children’s general and oral health/Socio-economic status/Education of parents/willingness to invest time	No policy suggestion was found
24	Al Garni et al. [43]	KSA	100/Convenience sampling/Interview/Bidding game	Implant	Gender/Income/Setting/Area of the missing/Perception toward oral health/Desire for implant	No policy suggestion was found
25	Edwards (thesis) [28]	Norway	948/General population/Face-to-face and telephone interviews/Open-ended	Publicly financed dental health care system	Age/Education/Income/Dental payments in the past year	No policy suggestion was found
26	Chau (Report) [37]	Hong Kong	1500/Convenience sampling/Questionnaire/Payment scale	Basic dental care plan	Income	Government can cover the secondary school students with different fees/(theme A)
27	Augusti et al. [64]	Italy	107/Convenience sampling Interview/Bidding-game	Implant-supported crown and the 3-unit fixed partial denture prosthesis	Perceived importance of oral care/Previous therapy	No policy suggestion was found
28	Atanasov et al. [56]	Bulgaria	111/Convenience sampling/Questionnaire/Open-ended	Treatment of molar: RCT + crown/Extraction and leaving a gap/Removable/Fixed bridge/implant	Household income	No policy suggestion was found
29	Srivastava et al. [65]	Canada	39/Convenience sampling/Questionnaire/Payment scale	Mandibular overdenture with two-implant	Age/Income/Know edentate or an overdenture wearing person	No policy suggestion was found
30	Moshkelgosha et al. [66]	Iran	200/Convenience Sampling/Questionnaire/Open-Ended	Orthodontic treatment	Education/income	Insurance policies for orthodontic treatment/(theme B)
31	Moshkelgosha et al. [44]	Iran	348/Convenience sampling/Interview/Payment scale	Different Orthodontic Brackets	–	No policy suggestion was found
32	Augusti et al. [67]	Italy	40/Convenience sampling/Questionnaire/Open-ended	Sonic and traditional brush	–	No policy suggestion was found
33	Vernazza et al. [4]	UK	503/Convenience sampling/Interview/Shuffled payment card	Treatment of molar teeth with Non-vital pulps	Income	No policy suggestion was found
34	Vernazza et al. [24]	UK And Germany	105/Convenience sampling/Questionnaire/Bidding card	Root caries prevention	No factor were significant predictors of WTP	No policy suggestion was found
35	Mckenna et al. [45]	UK	55/Convenience Sampling/Interview/Open–Ended With Payment Cards	Tooth replacement strategies: Removable Partial Dentures and functionally orientated treatment	Income levels/Previous Treatment experience	Changes are required to ensure that dental care is truly patient-centered/(theme B)
36	Kettlewell et al. [29]	Australia	1528/General population/Online questionnaire/DCE	Dental insurance	–	No policy suggestion was found
37	Dino et al. [30]	Italy	50/Convenience sampling/Questionnaire/Bidding game	Computerized anesthesia	Importance assigned by the patients to oral Care/Following recall programs	No policy suggestion was found
38	Fatani et al. [59]	KSA	171/Convenience sampling/Interview/Bidding game	Orthodontic treatment	Income	Orthodontists should educate the Public about occlusion/(theme C)
39	Nair et al. [55]	Singapore	83/Convenience sampling/Interview/Bidding game	Extraction/Filling and cleaning of teeth	Ethnicity/Age/Beliefs related to importance of oral health/Marriage status/Gender	No policy suggestion was found
40	Mubaraki et al. [53]	KSA	155/Convenience Sampling/Interview/Bidding Game	Space maintainer therapy for children	Family monthly income	No policy suggestion was found
41	Taschieri et al. [68]	Italy	103/Convenience sampling/Interview/Bidding game	RCT + crown or Extraction + implant	Education, employment	No policy suggestion was found
42	Sendi et al. [69]	Switzerland	16/Convenience sampling/Phone Interview/Bidding game	Implant retained complete dentures	–	No policy suggestion was found
43	Dino et al. [31]	Italy	50/Convenience sampling/Questionnaire/Bidding game	Computer-controlled Anesthesia	–	No policy suggestion was found
44	Sever et al. [38]	Croatia	265/Convenience Sampling/Questionnaire + Interview/DCE/Payment card in the pilot study	Dental care provided by a faculty member/Private dental care/Student-provided care	Age/Education	Findings may help policy-makers in delivering a care system Better suited to preferences of their patients etc./(theme B)
45	Ndambiri et al. [39]	Kenya	155/General population/Interview/Payment card	Fluoride removal from drinking water	Gender/Education/Household income/Living in own house/Type of water source/Perceived water quality/Distance to nearest water source/Payment vehicle/Household members experience of fluorosis/Age/Household size	Authorities could use the estimated mean and median WTP to benchmark their budget and water policy proposals for the removal of excess fluoride/(theme B)
46	Bratberg (thesis) [32]	Norway	438/General population/Questionnaire/TIOLI + Open-ended	Dental insurance	Age/Occupation/Income/Education	Implementation dental insurance may be possible/(theme B)
47	Nyamuryekung’e et al. [70]	Tanzania	1522/Convenience sampling/Interview/Open-ended	Extraction and filling	Age, Income/Outpatient Status/Experience	No policy suggestion was found
48	Vernazza et al. [46]	UK	401/Convenience sampling/Questionnaire/Payment card	Correction of malocclusions at different IOTN levels	Socioeconomic status/Sample recruited Center/Different IOTN Scenarios	No policy suggestion was found
49	Ramsay et al. [25]	UK and Scotland	667/General population/Questionnaire (online survey)/DCE	Oral hygiene advice/Periodontal instrumentation	–	No policy suggestion was found
50	Dalanon et al. [71]	Philippines	140/Convenience sampling/Questionnaire (online survey)/Payment scale	Fluoridization,/Prophylaxis/Extraction/Filling, Dentures/RCT/Oral cancer treatment, and orthodontic treatment	–	No policy suggestion was found
51	Walshaw et al. [26]	UK and Brazil	N = 200/Convenience sampling/Questionnaire/bidding format	Fluoride varnish	Income/Self-perceived need/recent Dental pain/child experience of restorations in last 2 years/Frequent attenders/Gender	No policy suggestion was found
52	Srivastava et al. [51]	Canada	317/General population/Internet-based questionnaire or telephone interviews/Bidding format	Dentures retained by implants	Gender/Age/Education/Income/Having dental insurance/Experience of implant/Having heard about implants/Self-perceived likelihood of edentulism	Desirability of including implant overdentures in insurance policies and public health programs/(theme B)

*WTP* willingness-to-pay, *NM* not mentioned, *YO* years old, *RCT* root canal therapy, *WATP* willingness and ability to pay, *DCE* discrete choice experiment, *IOTN * Index of Orthodontic Treatment Need

^a^Policy suggestions were categorized into three themes: (a) setting new tariff or subsidizing, (b) provision of the considered item by the public sector or its inclusion within the benefit package of public insurance due to population preferences, (c) promoting literacy

### Overview of the methodological aspects of WTP studies in oral health

Participant sample size broadly ranged from 16 to 1528 participants. A definite sample size calculation formula was reported in only eight studies [26, 38, 41–46]. Fifteen studies recruited samples from the general population [2, 6, 25, 27–29, 32–34, 39, 47–51] and others used the convenience sampling method. Twenty-two studies had response rates of more than 70% and 21 reported no rates of participation. Seven studies elicited WTP of parents for paediatric service without taking into consideration children preferences [26, 34, 36, 37, 46, 52, 53].

Data gathering was done by either an interview (28 studies) or a questionnaire (22 studies). Two studies used both methods simultaneously [38, 51]. To measure WTP, three studies used the Discrete Choice method [25, 29, 38], 11 studies used “open-ended” questions and one study reported no elicitation method in the original paper [54]. Twenty studies employed the “bidding-game”. The remaining studies employed the WTP elicitation methods: “Payment Scale” (n = 9), “Payment Card” (n = 6) and “TIOLI” (n = 2). Five studies used two elicitation methods simultaneously [18, 40, 47, 48, 55]. Twenty-eight studies declared the success rate of considered intervention as part of the scenarios presented to participants. Pretesting of the tool designed to elicit WTP amounts was done in 16 studies and, according to the authors, 5 studies were considered pilot studies [27, 32, 35, 37, 56]. Only eight studies in the design of their research explicitly reported any notion of preparation for exclusive bias prevention of the WTP method such as “starting-point” and “strategic” biases [6, 28, 32, 35, 47–49, 57].

### Overview of policy implications of WTP studies in oral health

Forty-one studies assessed participant characteristics influencing the stated amount of WTP. Of 40 studies 24 explored the association between “income” and WTP, and found a significant correlation between the two, among which only 6 studies made an adjustment of WTP results based on the different income groups. Furthermore, “age” (in 15 studies), “education” (in 13), “experience of receiving dental care” (in 7), “gender” (in 7) and “perceived importance of oral health” (in 4) showed WTP amounts to have a statistically significant correlation.

Sixteen articles (30%) proposed at least one suggestion pertaining to policymaking. These recommendations can be categorized in three main classes: (A) setting new tariffs or subsidizing some services for the whole population or special groups (e.g. exemption from patient payment)—7 recommendations [10, 33, 34, 37, 47, 50, 58]. (B) WTP as a direct indicative of participant demand, where 7 recommended direct provision of the service by the public sector or its inclusion within the basic benefit package of any form of public insurance in order to better fit the healthcare system to the patient preferences [32, 38, 39, 45, 45, 47]. (C) Government or professional communities promotion of oral health literacy by giving relevant information for public consumption in order to improve perceptions regarding oral health [care] and healthcare system characteristics—3 recommendations [24, 42, 59].

## Discussion

Although representative samples of the general population are recommended in WTP studies, especially for consequential allocation decisions, most studies have used convenience samples. More opportunities may have arisen to undertake participant face-to-face active communication interviews accompanied by more detailed information regarding the desired service(s). Some studies elicited parental WTP for paediatric services [26, 34, 36, 37, 46, 52, 53]. It is worth mentioning that these case results cannot be indicative of patient (child) perceptions, feelings and utilization of services which is an important aspect of the WTP method. Moreover, lack of standard sample size calculation formulas in dentistry has remained a common pitfall in WTP studies. Pretesting was not done in a majority of the studies (60%) which is consistent with the findings of the previous review study [22].

Cultural as well as health system attributes (including the predominant payment method) are two critical points to be undertaken in designing WTP studies in any setting. For example, although the ‘shuffled payment card’ is more likely to elicit true WTP amounts [16], it has been shown that the ‘bidding-game’ may be more suitable in developing countries [72]. In most of the studies, due to the unclear or inadequate details about contextual attributes in WTP reports, we are not aware of such considerations given in the design of these studies. Another limitation of this review was not identifying, by critical appraisal, the high-quality studies. According to the included studies in this review, the “bidding game” is the predominant method for eliciting WTP in the oral health field. This finding is consistent with previous oral health review articles [22].

In the majority of previous review article studies (57%) which analyzed the association between income and WTP amounts, have reported a statistically significant association; indicating a direct correlation between ‘ability’ and ‘willingness to pay’ [22]. A possible advantage of WTP studies that researchers can investigate is whether or not people in higher income groups tend to prefer one option more frequently than do those in lower income groups [7]. If WTP results are to be used as inputs for equitable resource allocation decisions, they should be adjusted according to the income differences, otherwise WTP estimations may lead to skewed resource allocations favoring the higher income levels of society [73]. Reporting the WTP amounts as a percentage of income is recommended for this purpose [74].

In all 13 studies which reported a statistically significant association between WTP values and education level, higher education was associated with higher WTP amounts. In the health field, the positive effect of education level on WTP values was previously shown in a literature review on WTP studies regarding diagnostic technologies in healthcare [75]. In a majority of the included studies which reported a statistically significant association between gender and WTP amounts (5 of 7), females exhibited higher WTP amounts This finding is consistent with the results of the previous review study [22].

The net benefit of a service to a society is evaluated by comparing it to its costs [11]. Only 31 studies of the studies reviewed have made such comparisons between benefits (WTP amounts) and real market prices or service costs.

Some authors presumed WTP as an amount which will be precisely paid by participants in real-life circumstances [10, 33, 34, 37, 47, 50, 58], whereas many studies have shown a significant overstatement in WTP figures due to hypothetical bias (potential difference between individual decisions in the real market and a hypothetical situation) [76–80]. Some studies have shown the potential of understatement in WTP amounts, especially in case of private goods and services [81].

A few authors have suggested that services be funded from public resources through either direct provision of that service by the public sector (government), or its inclusion within a benefit package through a national or social health insurance [32, 38, 39, 45, 45, 47]. Researchers should be aware of the part-whole bias (different values elicited for an item depending on whether it is valued solely, or as a part, in a more inclusive package) [82] in WTP studies which can magnify the utility of the service [83]. Even if the biases are fully prevented and we view WTP as a true proxy of population demand, still a suggestion needs further necessary criteria for it to be taken under consideration. For example, cost burden for a household, relative cost-effectiveness and socio-economic status of the potential consumers are just three of the important factors in deciding whether to finance a service under insurance coverage [84].

Some authors concluded from WTP results that health literacy should be improved within the population [24, 42, 59]. It seems that in these cases, the WTP method is reduced to an attitude assessment tool, whereas the WTP method could have more practical implications.

In this study Scopus and Web of science databases were not used and access to grey literature or personal communications was nonexistent. In future reviews of WTP studies in oral health, quality appraisal of conducted studies can unveil more details about the methodological aspects.

## Conclusions

In order to apply WTP studies to allocation purposes (as the benefit wing of cost–benefit analysis), there should be a consistency in the design of WTP studies [11], otherwise, WTP results are reduced to comparisons between desired services, when at least two choices have been considered, and this seriously limits the generalizability of the studies. Though no systematic and defined quality appraisal was done in this review, it appears that the majority of WTP studies in the field of oral health suffer from several deficits in some parts of the design such as sample size, representing samples, dealing with potential biases and pretesting. According to the current body of literature, the generalizability of oral health WTP results remains questionable, particularly in higher level decision making. It is felt that researchers who are interested in conducting WTP surveys for dental services need to pay more attention not only to the methodological aspects of the WTP studies, but to health policy knowledge as well, in order to conduct well-built studies in connection with meaningful and pragmatic policy considerations. It can be deliberated that accompanying research-minded policymakers, policy-minded researchers are also needed.

## Data Availability

All data generated or analysed during this study are included in this published article. All included articles in this review are available from NS on reasonable request.

## Abbreviations

WTP: Willingness-to-pay
CV: Contingent valuation
NM: Not mentioned
YO: Years old
RCT: Root canal therapy
WATP: Willingness and ability to pay
DCE: Discrete choice experiment
IOTN: Index of orthodontic treatment need
ISPOR: The leading professional society for health economics and outcomes research globally
